# Emotion processing and social participation following stroke: study protocol

**DOI:** 10.1186/1471-2377-12-56

**Published:** 2012-07-17

**Authors:** Clare L Scott, Louise H Phillips, Marie Johnston, Maggie M Whyte, Mary J MacLeod

**Affiliations:** 1 Rowett Institute, Greenburn Road, University of Aberdeen, Aberdeen, AB21 9SB, UK; 2 School of Psychology, Kings College, University of Aberdeen, Aberdeen, AB24 2UB, UK; 3 Health Science Building, Foresterhill Campus, University of Aberdeen, Aberdeen, AB25 2ZD, UK; 4 NHS Grampian, Foresterhill, Aberdeen, UK; 5 Rowett Institue of Nutrition and Health, Unviersity of Aberdeen, Greenburn Road, Aberdeen, Scotland, AB21 9SB, UK

## Abstract

**Background:**

The International Classification of Functioning, Disability and Health (ICF) defines participation as a person’s performance in life situations, including the size of social networks, and satisfaction with social contacts. Stroke survivors are known to experience a reduction in the number of their social networks and contacts, which cannot be explained solely in terms of activity limitations caused by physical impairment. Problems of emotional processing, including impaired mood, emotion regulation and emotion perception, are known to occur following stroke and can detrimentally influence many aspects of social interaction and participation. The aim of this study is to investigate whether emotion processing impairments predict stroke survivors’ restricted social participation, independent of problems with activity limitation.

**Methods/design:**

We aim to recruit 125 patients admitted to NHS Grampian with a confirmed diagnosis of stroke. All participants will be assessed on measures of emotion processing, social participation and activity limitation at approximately one month post stroke and again at approximately one year post stroke in order to assess change over time.

**Discussion:**

It is important to develop a greater understanding of the emotional factors which may underlie key social deficits in stroke recovery in an ageing population where stroke is one of the leading causes of severe, complex disability. This research may enable us to identify those who are risk of participation restriction and target them in the acute stroke phase of stroke so that adverse outcome is avoided and rehabilitation potential is fulfilled.

## Background

**Figure 1:**
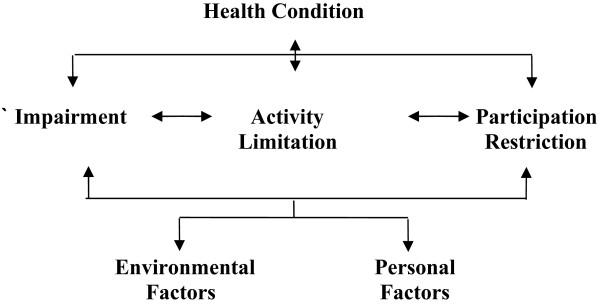
Graphic presentation of the ‘disability’ model of the International Classification of Functioning Disability and Health (World Health Organisation, 2001).

The International Classification of Functioning, Disability and Health (ICF) [1] provides a conceptual framework for examining the consequences of a health condition in terms of function and disability and illustrates how a health condition, such as a stroke, may impact upon an individual and their life (Figure 1).

The ICF has three health components, and a health condition may cause difficulty in any of these areas: The stroke may result in *Impairment* in the body function or structure, perhaps an inability to move a leg. Limitations in the individuals’ ability to execute an activity may occur resulting in an *Activity Limitation*, e.g. they may be unable to walk. Individuals may find themselves restricted in what they can participate in, e.g. perhaps they cannot participate in a social gathering, such as attending a walking club or even walking to church and as such will experience *Participation Restrictions* from this loss of social engagement in a life situation. These three health components of the ICF are further influenced by personal and environmental factors, which are unique to the individual, such as personality and beliefs, friends and family, as well as home environment. Crucially, within the ICF, difficulties can occur in one domain and not in another, so the relationship between the components of Impairment, Activity Limitations and Participation restrictions is not necessarily causal.

This current research investigates the health condition of stroke and looks at impairment in activity limitations and participation restrictions by examining the influence of impairments in emotion processing on social participation whilst controlling for activity limitations.

This research is informed by the literature in the fields of stroke, emotion processing and participation recovery in stroke, and this study protocol details the proposed methodology and analysis plan for this research.

### Stroke

Stroke is an increasingly common condition, and is the leading cause of severe, complex disability in the UK. More than 250,000 people live with disabilities caused by stroke. Economically, stroke care consumes an in excess of £2.8 billion of the UK National Health Service money each year in direct costs [2]. Within Scotland alone stroke care accounts for 5% of the total NHS budget [3]. Recovery from stroke is often associated with an intensive period of rehabilitation, with a focus on reducing levels of impairment and activity limitations. However, several studies investigating recovery from stroke over time, including a review of qualitative studies, have suggested that stroke survivors themselves do not tend to think of their aims in terms of overcoming physical limitations [4-6], but rather focus on achieving social integration back into community, family and work life.

There are many factors influencing stroke rehabilitation and recovery, and many barriers to the fulfilment of such a goal, in part due to the wide range of functions which stroke can impair, including motor control, language, memory and emotion processing. Given the ageing of the population, it is likely that incidence of stroke will continue to rise, and with more individuals surviving stroke, this is likely to be associated with important consequences for social interaction and the quality of life of both stroke survivors and their families.

### Social participation

The World Health Organisation has defined successful rehabilitation as reintegration of the individuals back into their social networks and participation in activities and the community [7]. Level of social participation is increasingly becoming regarded as a pivotal factor in successful rehabilitation [7]. The term social participation refers to the engagement in a variety of life situations, such as hobbies, recreation time, socialising, work, as well as the number and frequency of contacts an individual has. Yet stroke survivors are known to experience a reduction in social participation, as observed in reduced engagement in leisure and social activities, which cannot be explained solely by activity limitation [8,9]. Stroke survivors are also known to experience a decline in social networks and contacts [10]. Decreases in the quantity and quality of their social participation and interactions are key factors in levels of self rated quality of life [11]. Social participation has also been shown to be a key determinant of discharge destination from hospital suggesting that factors other than motor and cognitive limitations may be important in influencing post-stroke outcomes [12].

### Emotion processing

We aim to measure emotion processing in relation to three constructs of emotional experience and emotion skills; 1) Emotion Perception, 2) Emotion Regulation, and 3) Depression and Anxiety. Emotion processing has been recognised as a crucial component in social interactions between individuals [13] and as such difficulties in emotion processing may have implications for the individual’s ability to participate and interact in social situations [13,14]. First, impairment in the ability to accurately perceive emotions may impact on social participation as this skill forms an important part of our social interactions. Inability to judge whether someone is happy, sad, or angry, may result in confusing and dissatisfying social interaction, leading to increasing withdrawal from social situations [13,15]. Second, emotion regulation, specifically, this refers to the awareness, understanding and monitoring of our own emotions so that we may evaluate and influence which emotions we experience, when we experience them and crucially how strongly we experience them, in order to produce desired emotional states and goals. Finally, anxiety and depression are known to impact on the desire to engage in social interactions and participate in activities with other people [16]. In addition to this depression is known to affect how aware and sensitive we are to other people emotions [17] and is also associated with difficulties in emotion regulation [18]. In terms of stroke survivors, there is evidence to suggest that they are prone to all of these types of emotion processing impairment.

*Emotion perception* involves the identification of emotionally-salient information in the environment, and the decoding of these emotional states. This ability to decode emotional signals from faces and voices is considered to be an important component in the generation of emotional responses, emotional experiences and successful interaction in a social environment [14,19,20].

Numerous studies have investigated emotion perception ability in stroke survivors and demonstrated impairment in this ability [19-22]. However, to date, these have focused on focal unilateral brain lesions to investigate the role of each hemisphere of the brain in emotion perception abilities and have not examined the impact of such impairments on the individual’s life experience. Furthermore, there is little data available to evaluate the role of psychological factors on emotion perception following stroke. For example, stroke frequently results in depression, and this might influence aspects of emotion perception. Research with patients with major depression have demonstrated impairments in emotion perception [19,23] and this has been considered a factor in the progress and consolidation of depression. Montagne, et al. [19] found that depressed stroke patients were significantly more impaired in labelling emotions than both the controls and non-depressed stroke patients. The results of this study suggest that mood disorders may interact with stroke to influence emotion perception. As emotion perception ability has been demonstrated to be a key factor in social interactions and communication, the relationship between mood, emotion perception and social participation warrants further investigation in stroke survivors [14].

The definition of *emotion regulation* in the current research refers to the awareness, understanding and monitoring of our own emotions so that we may evaluate and influence which emotions we experience, when we experience them and crucially how strongly we experience them, in order to produce desired emotional states and goals. Individuals utilise different emotion regulation strategies in order to effectively control their emotions, enabling them to produce the emotional state which they wish to attain. The importance of emotion regulation has been highlighted in clinical populations, where impairments in this ability are correlated with the severity of their mental health condition [18,24,25]. Depression, anxiety, post traumatic stress disorder and schizophrenia are illnesses which have all been shown to impair emotion regulation, and although no direct link has been explored, these conditions are all associated with reduced social participation. Emotion regulation has also been identified as an important factor in psychological adaptation to chronic illness [26]. Successful emotion regulation has been associated with better perceived health, including psychological well-being and social functioning [27]. Although there has been no direct link explored between emotion regulation, as defined within this research, and stroke, specific brain networks are known to be responsible for emotion regulation [28]. As in the case of emotion perception, it is therefore possible that the lesion to the brain caused by the stroke may itself result in impaired emotion regulation. It is also possible that a reactive response to the stroke, as can be the case in depression, may also result in emotion regulation impairment. Here we explore the links between emotion regulation difficulties following stroke and social participation.

*Depression and anxiety* are relatively common following a stroke. A Cochrane review of literature suggested that at least one quarter of patients will experience depressive and anxiety disorders in the first year after onset of stroke [29]. Although the relationship between depression and the consequences of stroke is a complicated one, research has suggested that abnormal mood is correlated with impairments of physical, social and cognitive function, increased mortality and contributes to stress on carers [29-33]. Depression in stroke has been correlated with reduced social participation and contacts [16]. Less is known about anxiety following stroke and its impact on social participation.

### Aims and objectives

Therefore, this research aims to explore three different facets of emotion processing following stroke, exploring which difficulties in emotion processing occur and how these impairments impact on social participation in stroke survivors, while controlling for levels of activity limitation.

Participants will complete measures twice, at approximately one month post stroke and one year thereafter. This will allow the examination of the extent to which impairments in emotion processing are associated with social participation at both acute and chronic phases of stroke recovery. Also, by assessing participants longitudinally, direction of causation between emotion processing difficulties and social participation limitations can be explored.

The specific research questions which we will address are:

1) During the acute phase, approximately one month post-stroke, are deficits in emotion processing correlated with levels of social participation?

2) During the chronic phase, approximately one year post-stroke, are deficits in emotion processing correlated with social participation?

3) do emotion processing deficits in the acute phase of stroke predict social participation at one year post stroke which is not accounted for by activity limitation?

## Methods

### Design

This is a predictive study with a prospective design. Measures of emotion processing and social participation administered at baseline will be used to predict participation outcome at one year post stroke.

### Participants

A sequential cohort of 125 patients, approximately one month post stroke, will be recruited from the rehabilitation and acute stroke units, stroke outpatient clinics, and the NHS Grampian Stroke Register within Aberdeen Royal Infirmary and Woodend Hospital, Aberdeen. Potentially eligible participants are to be identified in collaboration with the acute stroke unit lead clinician.

### Inclusion criteria

Participants are included according to the following criteria: 1) a confirmed diagnosis of stroke, defined as the presence of symptoms persisting beyond 24 hours and/or a positive CT scan, one month previously; 2) no pre-existing neurological condition, psychiatric condition or chronic drug/alcohol abuse known to affect emotion processing; 3) no severe cognitive impairment (MMSE score >23).

### Measures

The following measures will be used at both the one-month post-stroke and one-year post-stroke assessments:

### Emotion processing measures

#### Emotion perception

There are several ways in which we gather cues as to other people’s emotions; from their facial expressions of emotions, from their voice through the emotional intonation within their speech. Previous research has suggested that the accurate perception and integration of all these cues are used in order to accurately infer what higher order emotions are being conveyed [13]. All three of these cues will be examined. To assess emotion perception in the visual modality the standardised Facial Expressions of Emotion: Stimuli and Test [34](Young, Perrett, Calder, Sprengelmeyer & Ekman, 2002) will be administered. This forced-choice paradigm requires participants to look carefully at static pictures of faces and choose which word best describes the emotion expressed in each picture from a provided list of six emotions; happy, sad, anger, disgust & fear.

In order to assess emotion perception ability in the auditory modality, a sub-test of the Florida Affect Battery [35] (Bowers, Blonder, Slomine & Heilman, 1996) will be administered. The emotion labelling task involves the presentation of one spoken sentence in an emotional tone. The participant then chooses from a list of words which emotion word best characterised the emotional intonation of the voice.

As we rarely view faces in isolation, or judge emotion on voice alone, a more ecologically valid measure of emotion perception has also been incorporated into the test battery. The Awareness of Social Inference Test [14], comprises 28 videoed vignettes of actors engaged in everyday situations in which they experience one of seven emotional states (happy, surprised, angry, sad, disgusted, anxious, and neutral) which the participant has to identify. The scripts in all cases are ambiguous in content. This measure is of particular importance as it has been established to have ecological validity with failure to recognise cues on TASIT translating into observable and reliable difficulties in spontaneous social situations [14], however as it has rarely been used in stroke populations before, this measure is administered in combination with the established measures described.

#### Emotion regulation

The methods by which emotions are regulated is dependent on several different factors, and so several areas of emotion regulation will be assessed, including the awareness and understanding of the emotion experienced; acceptance of the emotion; an ability to control impulsivity ensuring that one’s behaviour fits the desired goals even when experiencing negative emotions; and an ability to use flexible strategies to regulate emotional experience. Emotion regulation strategies will be assessed using the Difficulties in Emotion Regulation Scale (DERS) [36]. The DERS is a brief, 36-item, self-report questionnaire designed to assess multiple aspects of emotion dysregulation, including *nonacceptance* of emotions, *impulse* control of emotion, *awareness* of emotions, *strategies* of emotion regulation, ability to achieve desired *goals* and *clarity* of emotion.

#### Anxiety and depression

Depression and anxiety are common consequences of stroke, which have been shown to impact on other aspects of emotion processing as well as our desire to engage in social activities and contact. Two measures of depression and anxiety are to be administered. The first measure is the extensively used and validated Hospital Anxiety and Depression Scale [37] which has been shown to measure anxiety and depression, without confounding by physical symptoms. A further measure of depression the Centre for Epidemiological Studies Depression scale (CES-D) [38] will also be administered.

#### Social participation measures

Social Participation will be assessed in terms of several domains of engagement in social situations, including recreation, work, home activities, social aspects of life, as well as frequency of social contacts and networks. The Modified Functional Limitation Profile (mFLP) [39] will be administered. This is a hierarchical measure of participation, with 136 items giving scores across 12 categories. The categories established to assess social participation are those of; mobility, household, recreation, social interaction and work (Johnston & Pollard, 2001).

The World Health Organisation Quality of Life measure (WHO-QoL BREF) [40] is a multidimensional measure of quality of life, separated into four domains; physical health, psychological, social relationships and environmental. The WHO-QoL BREF has been shown to measure participation, and 80% of the WHO-QoL items have been demonstrated to tap some aspect of social participation [41] with 42% of those items purely measuring social participation. This measure specifically asks an individual about their *satisfaction* with their ability across the four domains.

Size of participants’ social networks will be assessed using the Lubben Social Network Scale (LSNS-18) [42]. This measure uses 18 questions which evaluate the size of different aspects of social network that are attributable to family ties, friendship ties and neighbourly ties. The LSNS-18 assesses the size of the respondent’s active social network (i.e., relatives or friends seen or heard from ≥1 times/month), perceived support network (i.e., relatives or friends who could be called on for help), and perceived confidant network (i.e., relatives or friends to whom the respondent could talk about private matters). Both the Lubben Social Network scale and the WHO-QoL BREF have been shown to measure participation as defined by the ICF [43].

### Activity limitation

The Modified Functional Limitation Profile (mFLP) [39] categories measuring activity limitation are those of ambulation, body care, alertness and communication.

### Impairment

Cognitive impairment will be assessed to provide a basic screen using the Mini Mental State Examination (MMSE) [44]. A score of less than 23 will result in the patient being excluded from the study.

### Procedure

Consecutive recruitment will be employed with potentially eligible participants identified from the stroke units and clinics. Participants are invited to participate in the study by letter from the lead clinician. Participants on the ward are given the letter, a participant information sheet and a reply form, and may then put their reply form in a box provided at the nurse’s station on the ward if they wish to participate. Participants discharged to home receive the same letter, information sheet and reply form posted to their home with a freepost envelope to post their reply form back to the researcher if they wish to participate. Participants who consent are required to complete two sessions, each session lasting approximately one and a half to two hours. The first session is to occur at approximately one month post stroke and the second session is to occur at approximately one year post stroke. All measures are administered at both time points. Participants can opt to split each session into multiple sessions should they wish to, to ensure that participants do not become fatigued. Participants can be assessed on the ward, in their home or at the University of Aberdeen.

### Ethical status and considerations

The North of Scotland Research Ethics committee have given a favourable ethical opinion for the study protocol, REC Number 08/S0802/8, and recruitment has commenced. Written informed consent is obtained from each participant as set out by the local ethical review board. No risk is associated with any of the measures to be used. All questionnaires being used are standardised and validated and have been used in previous studies with similar populations. Taking part in this study may involve minor inconvenience for the participants, in terms of spending time taking part in the assessments and in some instances travel to testing sessions. However, in a bid to alleviate the latter inconvenience participants will be given the option of the researcher attending their home or hospital ward. Any expenses that the participants incur will be reimbursed.

### Sample size

The sample size was calculated using a G-Power statistical sample size calculation. For F tests with a medium effect size, with an error probability of 0.05 and power of 0.95 the total sample required is 119.

### Analysis

Initially, a correlational analysis will be conducted on the measures from each domain of emotion processing to ascertain if the measures within that domain are highly correlated with each other. If this is the case the measures will be amalgamated. The same process will be conducted with the measures of social participation. Should the measures not be highly correlated an exploratory factor analysis will be conducted.

Regression analyses will be conducted to investigate whether the emotion processing measures predict social participation measures, controlling for activity limitations. The final research question was whether emotion processing deficits in the acute phase of stroke predict social participation at one year post stroke which is not accounted for by activity limitation? To address this, hierarchical regression analyses will be conducted, predicting social participation at second assessment from concurrent activity limitation in the first step and emotion processing from the acute phase of stroke entered in the next step of the regression.

## Discussion

### Potential implications of work

This project will address a number of health care service priorities, including stroke, mental health, ageing and quality of life. This research has the potential to improve knowledge of the effects of stroke on mood and emotion perception, as well as enhancing our knowledge of the relationship between emotion processing constructs and participation outcome. Improving our understanding of the emotional factors which may underlie key social deficits in stroke is important to inform models of functioning and disability following stroke, as factors other than activity and cognitive limitations may be important in influencing post-stroke outcomes.

The results from these studies will be of interest not only to the academic community with an interest in ageing and health outcomes, but also to both medical groups and patient support groups with an interest in stroke. In the longer term, the results from this study may influence future practice within rehabilitation settings. If emotion processing difficulties predict participation levels and activity limitation, then those individuals could potentially be targeted for intervention in the acute stroke phase, ensuring that rehabilitation potential is fulfilled. Interventions have previously been successful in improving recovery in stroke patients. A randomised controlled trial of an intervention to increase patients’ perceived control over their recovery resulted in better recovery from activity limitations [45]. Interventions targeting emotional states in the acute phase of stroke have been found to improve quality of life outcomes [30]. This research is also important in combining theoretical and applied scientific approaches from health psychology and cognitive neuroscience to important health problems.

## Competing interests

The authors declared that they have no competing interest.

## Authors’ contribution

CS, LP, and MJ developed the initial study design and undertook the sample size calculations. All authors further developed the study protocol. MJM facilitates access to participants and recruitment. CS is responsible for co-ordinating participant recruitment, and project management under the supervision of LP, MJ, MW and MJM. All authors contributed to writing this manuscript for submission. All authors read and approved the final manuscript.
